# Challenges to the Effectiveness and Immunogenicity of COVID-19 Vaccines: A Narrative Review with a Systematic Approach

**DOI:** 10.3390/vaccines13080789

**Published:** 2025-07-24

**Authors:** Alexander A. Soldatov, Nickolay A. Kryuchkov, Dmitry V. Gorenkov, Zhanna I. Avdeeva, Oxana A. Svitich, Sergey Soshnikov

**Affiliations:** 1 Scientific Centre for Expert Evaluation of Medicinal Products of the Ministry of Health of the Russian Federation, 8/2 Petrovsky Boulevard, Moscow 127051, Russia; patosold@mail.ru (A.A.S.); avd-cytok@yandex.ru (Z.I.A.); 2 CEG PharmDev DOO, 21000 Novi Sad, Serbia; nkryuchkov@clineg.com; 3 I. Mechnikov Research Institute of Vaccines and Sera, 5A, Malyi Kazenny Lane, Moscow 105064, Russia; 4 Public Health Division, School of Health Sciences, Central Michigan University, Health Professions Building, CMU, Mount Pleasant, MI 48859, USA

**Keywords:** COVID-19, SARS-CoV-2, vaccines, vaccine efficacy, vaccine immunogenicity, vaccination, immunization

## Abstract

The COVID-19 pandemic accelerated the rapid development and distribution of various vaccine platforms, resulting in a significant reduction in disease severity, hospitalizations, and mortality. However, persistent challenges remain concerning the durability and breadth of vaccine-induced protection, especially in the face of emerging SARS-CoV-2 variants. This review aimed to evaluate the factors influencing the immunogenicity and effectiveness of COVID-19 vaccines to inform future vaccine advancement strategies. A narrative review with systematic approach was conducted following PRISMA guidelines for narrative review. Literature was sourced from databases including PubMed, Embase, and Web of Science for studies published between December 2019 and May 2025. Encompassed studies assessed vaccine efficacy, immunogenicity, and safety across various populations and vaccine platforms. Data were collected qualitatively, with quantitative data from reviews highlighted where available. We have uncovered a decline in vaccine efficacy over time and weakened protection against novel variants such as Delta and Omicron. Booster doses, specifically heterologous regimens, improved immunogenicity and increased protection. Vaccine-induced neutralizing antibody titers have been found to correlate with clinical protection, although the long-term correlates of immunity remain poorly defined. The induction of IgG4 antibodies after repeated mRNA vaccinations raised concerns about potential modulation of the immune response. COVID-19 vaccines have contributed significantly to pandemic control; however, their efficacy is limited by the evolution of the virus and declining immunity. Forthcoming vaccine strategies should focus on broad-spectrum, variant-adapted formulations and defining robust comparisons of protection. Recognizing the immunological basis of vaccine response, including the role of specific antibody subclasses, is fundamental for optimizing long-term protection.

## 1. Introduction

The rapid development of COVID-19 vaccines represents an unprecedented scientific achievement that has significantly mitigated severe disease and mortality during the SARS-CoV-2 pandemic [1,2,3]. These vaccines—spanning mRNA, viral vector, inactivated, and protein subunit platforms—demonstrated initial efficacy of 52–95% against symptomatic infection and 74–100% against severe outcomes [4,5,6]. Nevertheless, two interrelated challenges threaten long-term vaccine utility.

First, waning and variant-susceptible immunity: progressive decline in neutralization titers (e.g., BNT162b2 VE: 91.3% → 60% at 6 months) [7,8], and immune evasion by Variants of Concern (VOCs), particularly Omicron sublineages (e.g., 37% VE reduction vs. ancestral strain) [9,10]. Second, unresolved immunological mechanisms: incomplete definition of durable correlates of protection beyond transient antibody titers [11], and emergence of IgG4 subclass dominance after repeated mRNA vaccination (≤19.27% of anti-spike IgG) with uncertain clinical implications [12,13].

Heterologous boosting and variant-adapted vaccines offer promising approaches to enhance protection [14,15], yet key questions persist regarding optimal platform combinations and dosing intervals, functional consequences of vaccine-induced IgG_4_ switching, and standardization of cross-protective immune correlates.

This narrative review presents current evidence to quantify efficacy attenuation against VOCs, evaluate the immunogenicity of homologous versus heterologous regimens, analyze the immunological drivers of waning protection, and propose frameworks for the design of next-generation vaccines. We examined the immunological, clinical, and public health aspects of COVID-19 vaccine effectiveness in the context of evolving viral variants and immune response mechanisms.

## 2. Materials and Methods

**Figure 1:**
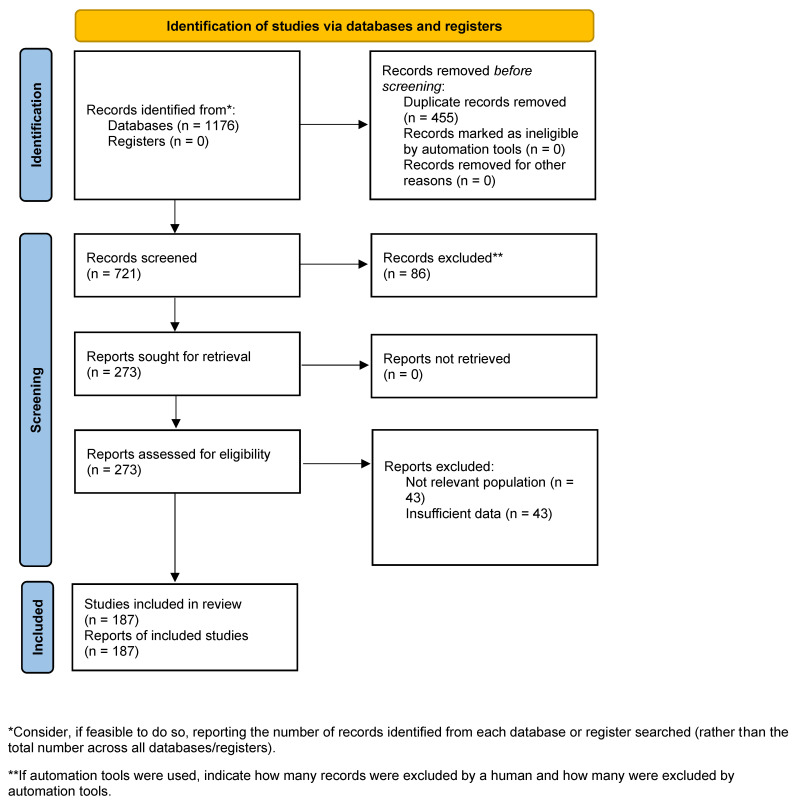
PRISMA 2020 flow diagram.

This review was conducted as a narrative review with a systematic approach, aiming to synthesize and critically analyze current evidence on the challenges to the effectiveness and immunogenicity of COVID-19 vaccines. The review process followed established guidelines for systematic and narrative reviews, adhering to the PRISMA (Preferred Reporting Items for Narrative Reviews) framework where applicable (Figure 1). The methodological approach was designed to ensure comprehensive coverage, transparency, and reproducibility.

A comprehensive literature search was performed across multiple electronic databases, primarily PubMed, Embase, Web of Science, Scopus, Cochrane Library, and relevant preprint servers. The search covered publications from 1 December 2019, to 1 May 2025. The search strategy combined Medical Subject Headings (MeSH) and free-text terms to maximize sensitivity and specificity. The following keywords and Boolean operators were used:

“COVID-19” OR “SARS-CoV-2” OR “coronavirus disease 2019”

“vaccine” OR “vaccination” OR “immunization”

“vaccine efficacy” OR “vaccine effectiveness” OR “immunogenicity”

“booster” OR “heterologous” OR “prime-boost” OR “neutralizing antibodies” OR “IgG4”

“variant” OR “Omicron” OR “Delta” OR “variant of concern (VOC)”

“adverse events” OR “safety” OR “reactogenicity”

The search was supplemented by screening reference lists of relevant articles and systematic reviews to identify additional studies. Only articles published in English were included.

Key studies representing diverse approaches were selected based on relevance.

Original research (randomized controlled trials, cohort studies, case-control studies, cross-sectional studies), systematic reviews, or meta-analyses focused on COVID-19 vaccine efficacy, effectiveness, immunogenicity, or safety. Studies involving human participants of any age or risk group, including special populations (e.g., older adults, people with autoimmune diseases, immunocompromised individuals). Articles reporting on primary, booster, homologous, or heterologous vaccination regimens. Studies evaluating outcomes such as breakthrough infection, seroconversion, neutralizing antibody titers, adverse events, or vaccine effectiveness against variants.

Exclusion criteria included non-original articles (e.g., editorials, commentaries), animal studies, and articles lacking extractable data on relevant outcomes.

All identified records were screened independently by two reviewers. Titles and abstracts were first assessed for relevance, followed by full-text review. Discrepancies were resolved through discussion or consultation with a third reviewer. Data extracted included study design, population characteristics, vaccine type, immunization regimen, outcomes (e.g., efficacy, immunogenicity, safety), and key findings.

Given the narrative nature of this review and the heterogeneity of study designs, populations, and outcomes, this narrative synthesis draws on emerging patterns. Special emphasis was placed on the impact of emerging variants, booster strategies, and the immunological mechanisms underlying vaccine responses.

After removing duplicates and screening titles and abstracts for relevance, 273 full-text articles were selected for review. The final reference list included 185 studies, reflecting the breadth of the literature on COVID-19 vaccine challenges. Notably, recent systematic reviews and meta-analyses included in this review have incorporated up to 50 studies each, with some analyses covering over 775,000 individuals. The review encompasses evidence on mRNA, adenoviral vector, inactivated, and protein subunit vaccines, as well as heterologous and homologous booster regimens.

## 3. Results

### 3.1. Vaccine Efficacy and Effectiveness

Scientific research regarding the development of vaccine production methods began in the mid-19th century. Various techniques were used to produce vaccines that were designed to prevent a wide range of infectious diseases. The global health crisis associated with the SARS-CoV-2 pandemic has provided a significant stimulus for the development of vaccines designed to combat the infection. These vaccines were rapidly authorized for use and subsequently made available for mass immunization worldwide. At the end of 2022, more than 50 COVID-19 vaccines had been approved by regulatory authorities in various countries worldwide (based on the authorization requirements regarding the marketing of medicines and drug products in emergencies), and more than 240 vaccines were subjected to preclinical and clinical trials [16]. The World Health Organization (WHO) had prequalified 11 COVID-19 vaccines by November 2023, including two mRNA vaccines (Comirnaty and Spikevax), four virus vector vaccines (Vaxzevria, Covishield, Ad26.COV2.S and Convidecia), three inactivated-virus vaccines (Covilo, CoronaVac and Covaxin) and two protein subunit vaccines (Nuvaxovid and Covovax) [17].

It should be taken into account here that assessing the efficacy of these vaccines, with rare exceptions, is a complex and lengthy process. Moreover, previously obtained efficacy results may be adjusted afterward due to the emergence of more reliable and relevant results from clinical and epidemiological studies, including changes in the parameters of the target population and the infectious agent over time.

Vaccines may form protection against loss of life (death), severe disease, hospital admission, transmission of infection, and other adverse events. At the same time, all these types of efficacy are influenced by many factors, such as sex, age, concomitant diseases, pathogen characteristics (variants, subvariants, strains), current anti-infectious measures, etc. Vaccine studies use many different endpoints to determine efficacy depending on the pathogen, the infection’s consequences, and the epidemiological process’s time course. For example, the outcome of randomized controlled trials (RCTs) is often formulated as a proportional reduction of disease incidence in groups of vaccinated volunteers and a control group (not vaccinated), with an assessment of the parameter VE—the disease incidence reduction degree associated with vaccination [18]. Vaccine efficacy assessment may also include an analysis of the reduction in the infection transmission rate (i.e., evaluation of the formation and strength of sterilizing immunity), the severity of clinical manifestations and disease duration, as well as changes in the immune response and degree of severity [18,19].

The efficacy of vaccine prevention identified in clinical studies does not always accurately reflect the actual effectiveness in various subpopulations [20]. For example, the efficacy of the rotavirus vaccine in children in low- and middle-income countries is lower than that observed in high-income countries [21]. A live rotavirus vaccine efficacy study involving more than 69,000 subjects, primarily from high-income countries, demonstrated 95% efficacy with respect to severe disease in vaccinated individuals [22]. According to the meta-analysis results in the paper by Soares-Weiser K. et al., the analysis of 34 vaccine clinical trials (181,009 study participants) failed to establish the vaccine’s effect on preventing a fatal outcome of rotavirus infection [23]. Based on a comprehensive analysis of post-registration experience with the vaccine, which prevents rotavirus infection, a significant reduction in mortality was observed in patients whose primary symptom of the disease was diarrhea [24]. However, the immunization schemes play a very important role and were also discovered.

### 3.2. Primary and Booster Immunization Schemes

Following SARS-CoV-2 vaccine development and granting of marketing authorization, the high relative efficacy of vaccines was demonstrated at 50–93%; the ability of the vaccines to prevent the disease from increasing in severity was shown to be 74.6–100%, thereby significantly reducing hospital admission and mortality rates from COVID-19 (Table 1). 

Following the initiation of an active COVID-19 population vaccination campaign, it was observed that the protective effects of the vaccines weakened over time after the primary immunization series [8,34]. For instance, the disease (of any severity) prevention efficacy of the Comirnaty vaccine (BNT162b2) decreased from 90% to 60% six months after vaccination [5]. At the same time, reinfection episodes were observed among fully vaccinated individuals and those who recovered after COVID-19 [35]. In particular, antibody-positive recovered subjects had 89% protection against reinfection [36], and the vaccine efficacy against reinfection ranged from 50% to 95% [37].

Most studies on the efficacy of mRNA vaccines have shown that they can protect against severe disease and a fatal outcome for only 6–8 months [1,7]. This is primarily determined by the nature of the immune response to SARS-CoV-2 infection. Furthermore, the high mutability of the virus also plays an important role. New variants, sublineages, and strains that emerged during the SARS-CoV-2 pandemic can escape the neutralizing antibodies induced by primary vaccination or previous episodes of the new coronavirus infection [9,38]. Antibodies to early SARS-CoV-2 strains have shown significantly reduced cross-reactive neutralizing activity against the Delta and Omicron variants, leading to an increase in reinfection rates in many regions of the world [39,40,41].

The well-known, relevant technique to enhance vaccine effectiveness is administering additional (booster) immunization doses. This understanding has led to the development of optimal and sustainable vaccination schemes. Research and discussion then began to determine and establish the most suitable period between administering the primary and booster series, as well as the efficacy of homologous and heterologous schedules.

Studies have shown that booster doses increase and prolong vaccine efficacy. In particular, a Phase III clinical trial (NCT04955626) demonstrated that administering a third dose of the BNT162b2 vaccine 10.8 months after the second dose provided 95.3% relative efficacy during a follow-up period of 2.5 months [42]. The clinical study showed that a booster vaccination of the Ad26. The COV2.S vaccine, administered two months after the initial dose, significantly increased vaccine efficacy, demonstrating a 75% reduction in infection rates and ensuring that the disease did not increase in severity in 100% of cases [43].

According to the results of the first clinical trial evaluating the effectiveness of the booster ChAdOx1 nCoV-19 vaccine (AZD1222), a second dose was recommended 8–12 weeks after the initial vaccination [3,44]. Another study demonstrated that the longer the period between the primary and booster administrations, the more intense the humoral immune response to the ChAdOx1 nCoV-19 vaccine (AZD1222) [45]. The development of the immune response was assessed following a single administration of the ChAdOx1 nCoV-19 (AZD1222) vaccine, as well as after the second administration (8–12, 15–25, or 44–45 weeks after the first administration) and after the third administration (28 weeks after the second administration). The study demonstrated that the antibodies’ geometric mean titers (GMTs) approximately 320 days after the single administration amounted to 66 ELISA units (EU). Interestingly, the level of antibodies was higher with a longer interval between the first and second administrations (GMTs of antibodies with the subsequent administration of the drug product after 8–12 weeks was 923 EU, after 15–25 weeks—1860 EU, and after 44–45 weeks—3738 EU) [45]. For those individuals who received the third administration of the drug 28 weeks after the second dose, GMT reached 3746 EU. Despite numerous clinical studies substantiating the booster administration of the ChAdOx1 nCoV-19 vaccine (AZD1222), manufacturers did not submit applications to regulatory authorities to secure official approval for administering the booster vaccine doses. Multiple clinical and observational studies of various COVID-19 vaccines have failed to identify the optimal period between the primary and booster administrations relevant for all or most such vaccines. We also made a comparison with the other types of immunization schemes below.

### 3.3. Heterologous Immunization Schemes

The heterologous immunization approach was studied to increase the immunogenicity, effectiveness and safety of the COVID-19 vaccination schedules and diminish the chances of drug supply issues during mass immunization campaigns. Thus, mRNA vaccines were suggested as a second dose for people vaccinated with a single dose of adenovirus-based ChAdOx1. Studies have demonstrated that administering a primary dose of ChAdOx1 in adults, followed by a dose of heterologous mRNA vaccine (BNT162b2 or mRNA-1273), results in a more robust humoral immune response compared to the homologous ChAdOx1 vaccine scheme [46,47,48,49,50]. A phase II clinical trial (Com-COV2) compared volunteers primed with ChAdOx1 or BNT162b2 followed by a homologous vaccine, mRNA-1273, or NVX-CoV2373, as a second dose. This study demonstrated that heterologous immunization schedules induced a more robust synthesis of anti-spike IgG antibodies compared to homologous schedules. The administration of the heterologous mRNA-1273 vaccine resulted in a higher humoral anti-spike IgG response than that achieved by the homologous vaccination in individuals who had received the primary immunization with ChAdOx1 or BNT162b2 [51]. Similarly, a French observational study of actual clinical practice, which enrolled healthcare professionals, showed that the heterologous combination of BNT162b2 and ChAdOx1 provided more robust protection than vaccination with the homologous vaccine (BNT162b2) [14]. Moreover, it was found that following the administration of the heterologous vaccine ChAdOx1, after priming with CoronaVac, the synthesis of specific antibodies against the RBD domain of S protein remained eight times higher than with the homologous vaccine (CoronaVac) [52].

Booster administration of mRNA vaccines (BNT162b2 and mRNA-1273) significantly increases the titers of neutralizing antibodies [14]. In some countries, such as the UK and Australia, mRNA vaccines (BNT162b2 and mRNA-1273) are administered as a booster dose to individuals who have received the ChAdOx1 vaccine. The US Food and Drug Administration (the US FDA) authorized the use of Ad26.COV2.S vaccine according to homologous and heterologous booster schedules before the later suspension of its use due to concerns about rare cases of a blood clotting syndrome [53]. It should be noted that Ad26.COV2.S is not approved for administration in children and adolescents.

Theoretically, activating local immunity in the upper respiratory tract (mucosal immunity) should increase protection against infection by preventing the virus from contacting the tissues of the respiratory system. Considering this, Convidecia vaccine developers proposed an orally inhaled aerosol for booster vaccination. Clinical studies have shown that the Convidecia vaccine oral booster induced a more robust immune response in patients initially vaccinated with two doses of CoronaVac [54]. Immunization schemes play a vital role and can differ, but we must also consider other approaches to immunization, such as boosting.

### 3.4. Booster Vaccination Against New Strains of the Virus

Many countries started using vaccine boosters in the last quarter of 2021 when the Delta variant was predominant [55,56]. However, by the beginning of 2022, the Omicron strain had replaced all previous variants of SARS-CoV-2 worldwide. Among those vaccinated with the BNT162b2 homologous booster, the efficacy against the Delta and Omicron strains remained at 88–93% and 76%, respectively, and the efficacy against an increase in the severity of the disease induced by the Delta and Omicron variants was 97% and 89%, respectively [10]. Reports from the US Centers for Disease Control and Prevention (CDC) indicate that in cases of vaccination with boosters of a homologous mRNA vaccine (BNT162b2 or mRNA-1273), the efficacy against the Delta and Omicron lineages is 93% and 67%, respectively. The efficacy against hospital admissions caused by the Delta and Omicron strains was 94% and 90%, respectively [57].

Despite the high efficacy of booster vaccination against new strains, following the emergence of the Omicron strain, a breakthrough in the infection rate was observed. In particular, COVID-19 cases rose sharply in people vaccinated with BNT162b2 or mRNA-1273 [58,59,60,61,62,63]. The third administration of BNT162b2 or mRNA-1273, administered less than six months after the second administration induced protection against the Delta and Omicron strains. Higher levels of protection were recorded against the Delta strain compared to the Omicron strain [57].

In people vaccinated with ChAdOx1, administered the heterologous BNT162b2 vaccine as a booster, the efficacy against Delta and Omicron infections was 94% and 71%, respectively [64]. A similar study in Brazil compared four booster doses (ChAdOx1, BNT162b2, Ad26.COV2.S, or CoronaVac) in recipients who were primarily vaccinated with the CoronaVac product. At the same time, heterologous boosters were shown to induce high concentrations of neutralizing antibodies in pseudovirus tests and virus neutralization studies with the Delta and Omicron variants [65]. Although strains of the Delta and Omicron variants can cause breakthrough infections in people who have undergone timely revaccination, the viral load in these individuals is lower, and COVID-19 symptoms are less pronounced [66,67].

Revaccination is especially important for vulnerable and high-risk groups. In particular, it has been demonstrated that older adults require an additional booster, such as BNT162b2 or mRNA-1273, to achieve sufficient protection against Delta and Omicron infections [68,69]. In January 2022, Israel started offering a second booster vaccination to older adults and people with compromised immunity [70,71]. A retrospective study in Israel found that a second booster dose of BNT162b2 reduced hospital admissions and fatal outcomes associated with COVID-19 in people over 60 compared with a single booster dose [72]. In March 2022, the US FDA also began recommending a second booster dose of the mRNA vaccine in older adults and people with comorbid conditions [73,74].

In studies evaluating the efficacy of booster injections against the Delta and Omicron variants, the highest efficacy rate was observed in individuals who had already been infected with the virus. In particular, the work of Zheng et al. demonstrated that booster immunization triggers the synthesis of high levels of neutralizing antibodies, reduces the number of clinical symptoms associated with COVID-19, and leads to faster virus clearance in patients infected with the Omicron variant [75]. Hybrid immunization (natural infection and vaccination) induces, on average, higher levels of neutralizing antibodies than immunization itself. It is supposed that the higher efficiency is because, during the primary infectious inflammation, an immune response is induced against the invariant region of the SARS-CoV-2 virus—N-protein, which is almost not subject to mutations and remains constant since mutations mainly affect the S-protein, which in turn can provide a more pronounced and broad adaptive cell-mediated immune response.

Thus, during the post-vaccination period, the efficacy of vaccines decreases over time, while the additional administration of vaccines (booster) significantly increases vaccination efficacy. Moreover, heterologous vaccine regimens, used during both the primary and booster administration stages, are more immunogenic and efficient than homologous immunization schedules. Moreover, even booster administration of heterologous vaccines based on the “Wuhan strain” significantly increased the efficacy of the preventive immunization schedule against the Delta and Omicron strains. Even greater efficacy was demonstrated in those cases of hybrid immunization (vaccination of people who previously had the disease).

### 3.5. Particularities of the Immune Response to SARS-CoV-2 Infection and COVID-19 Vaccination

The initiation of a specific immune response to vaccine antigens, accompanied by the formation of immune memory, determines the efficacy of vaccination. In most infections, immune defense is due to activation of the humoral component of adaptive immunity. Therefore, the production of specific, neutralizing antibodies or specific antibodies with neutralizing activity is a vital indicator of an efficient immune response to vaccination (a surrogate measure of vaccine efficacy). A vaccine is believed to be efficient if it causes the same or similar immune response as during an appropriate infectious inflammation.

COVID-19 is a new disease, and the mechanisms of infection and vaccination have not yet been completely understood. A risk group (medical workers) study showed that 0–5 days after the onset of COVID-19 clinical symptoms, only 37.5% of patients had specific IgM antibodies and 60.2% had IgG antibodies. Within 25 days after the onset of clinical symptoms, an increase in the seroconversion of IgM and IgG antibodies was observed. A decrease in seropositive levels in individuals was observed 36–95 days after the development of symptoms, more pronounced for IgM (17.9%) than for IgG (58.9%) antibodies. Overall, it was found that 83.6% of IgM-positive and 32.9% of IgG patients, respectively, were antibody-positive within the first month of the onset of clinical symptoms, and then became antibody-negative in the third month after developing clinical symptoms [76]. Another study showed a trend toward a decrease in both IgG and IgM antibodies, compared with antibody levels at hospital discharge, within seven months after the onset of symptoms [77].

The efficiency of COVID-19 vaccines is promoted by neutralizing antibodies (NAbs). Dispinseri S. et al. showed that NAb levels correlate with survival and virus control in the infected patients [78]. A lack of NAb synthesis early after the onset of the disease is, to a high degree of accuracy, correlated with mortality and delayed virus control, to a greater extent than with NAb levels. Almost all patients developed neutralizing antibodies (NAbs) by the fourth week of infection. At that, severely ill patients had higher peak titers, faster increases of antibody levels, and higher Nab titers than patients with mild disease [79,80,81]. Hospital patients have higher NAb titers than patients with mild disease and asymptomatic patients (with a titer below the detection limit in half of the cases) [64]. However, in infected individuals, the levels of NAb and IgG are significantly reduced after a year of observation [75].

A perfect vaccine should provide efficient long-term protection, maintain a tangible safety profile, and remain affordable and readily available to all population groups [81]. Initial studies of COVID-19 prevention vaccines demonstrated that vaccines on different technological platforms exhibited varying abilities to initiate the synthesis of neutralizing antibodies and, accordingly, had different rates of protection against infection (Table 2).

The changes in immunological indicators are usually considered as part of the secondary endpoints of vaccine efficacy and immunogenicity (surrogate efficacy outcomes). Regarding immunogenicity analysis, it is essential to identify the correlation between the intensity of immune responses and other immune response characteristics with vaccine-induced protection against relevant infectious diseases. Such endpoints are referred to as Immune Correlates of Protection (ICPs) [91].

At the same time, many years of experience developing ICPs show the complexity of their justification. In particular, a study involving individuals infected with the influenza virus established a correlation between the clinical symptoms of the disease and the levels of immunoglobulins in the serum and mucous membranes, as well as indicators of T-cell immunity, with the conclusion that certain parameters of immunogenicity are ICPs [92,93,94,95,96]. However, other studies after vaccination with inactivated or live attenuated vaccines, followed by influenza infection, did not prove protection-related immune correlates [97]. Regarding influenza infection, a hemagglutination inhibition (HAI) titer of 1:40 is considered to provide 50% protection against influenza [98]. Other studies suggested a titer ranging from 1:17 to 1:110 [99,100]. Nevertheless, a 1:40 titer was justified by immunogenicity assessment determined over many years using data from a standardized HAI assay and applied to serological samples obtained from human challenge infection and cohort studies [101].

Since the start of mass COVID-19 vaccination, efforts have been made to determine and justify the levels of anti-SARS-CoV-2 antibodies produced in response to immunization, correlating these levels with vaccine efficacy and disease outcomes. In a study that enrolled healthcare workers (risk group), specific antibodies were shown to be a good predictor of a lower infection risk. The authors suggest that antibody levels can serve as a marker of protection against the new coronavirus infection [102]. However, the study did not compare antibody levels between infected and uninfected individuals, which does not allow for the objective justification of ICP indicators. In phase III clinical studies, Khoury D.S. et al. and Earle K.A. compared 7 COVID-19 vaccines and showed that vaccine-induced neutralizing antibodies strongly correlate with the protective effect [11,103]. This study has the disadvantage of using non-standardized methods to assess specific antibodies. Additionally, the studies were conducted in various countries at different times. Therefore, the vaccinated individuals and the predominant virus strains in these studies differ, potentially distorting the efficacy analysis results.

Gilbert P.B. et al. and Feng S. et al. published studies to justify ICPs for COVID-19 vaccines developed by Moderna and AstraZeneca [104,105]. They showed that peak levels of antibodies specific to binding protein and neutralizing antibodies induced by the mRNA-1273 and ChAdOx1 vaccines were sound predictors of protection against the symptomatic disease. The findings can be used to predict efficacy when modifying the vaccination regimen or frequency of booster vaccination. Since only two drugs were evaluated in these studies, whether the results can be extrapolated for other vaccines remains unclear. Overall, sufficiently reliable evidence of the relationship between neutralizing antibody titers in vitro and protection against SARS-CoV-2 in vivo is still unavailable [106].

A connection between the disease outcomes and the presence of neutralizing antibodies (NAbs) in infected individuals was observed. Still, at the same time, no correlation was established between the concentration of neutralizing antibodies or their titer and the outcome, characteristics, or severity of the disease. It was also confirmed by studies of convalescent plasma medicines and monoclonal antibody drugs used in the treatment of COVID-19. In developing these therapeutic approaches, the authors have failed to define NAb titers in the plasma of convalescents or concentrations of monoclonal antibodies that correlate markedly with drug efficacy. Additionally, it was not possible to detect a dose-dependent effect for monoclonal antibody medicines across a wide dose range [107]. Moreover, some studies showed a deterioration in the patient’s condition in the presence of severe disease following the administration of the drug product based on specific antibodies. Therefore, monoclonal antibody drugs are administered only to patients with mild to moderate new coronavirus disease [108,109].

Bartsch Y.C. et al. studied the neutralizing (blocking, protective) activity of antibodies produced in response to SARS-CoV-2 infection. The authors showed that the protective activity of antibodies manifested itself at a specific antibody threshold level. The specific antibodies produced in individuals with asymptomatic or mild COVID-19 have no neutralizing properties. In individuals with severe disease, both humoral and cellular protective immune responses are formed during the production of antibodies with a concentration of at least 0.1 μg/mL [110]. Individuals with high titers of specific IgG are distinguished by broad and strong RBD-, N- and S-specific humoral immune reactions involving antibodies of different subclasses and isotypes and a pronounced innate immune response.

Although COVID-19 vaccines can induce high titers of neutralizing IgG and IgA class antibodies against the SARS-CoV-2 spike protein, they only offer temporary protection against the SARS-CoV-2 infection [1,111,112,113]. The high rate of reinfection caused by the Omicron strain suggests that current immunization schedules provide no sterilizing protection [114].

### 3.6. Characteristics of Specific Antibodies Produced in Response to Vaccination

Several studies have been conducted to determine the causes of post-vaccination reinfection, with a focus on assessing the avidity of neutralizing antibodies. Avidity is established during affinity maturation, and failure to achieve high IgG avidity can lead to a lack of protective immunity against infection and disease. However, during the development of an immune response to SARS-CoV-2, avidity maturation is incomplete, which is followed by a decrease in the serological response [115]. Due to the high degree of variability in the IgM and IgG responses to SARS-CoV-2, kinetic patterns of Acute and past infections cannot be differentiated by measuring IgM and IgG antibodies exclusively [116]. Among other things, the lack of high avidity of specific antibodies increases the frequency of reinfection, hampering the achievement of herd immunity [115]. The avidity is significantly higher in patients requiring hospital admission than outpatients, possibly due to a higher viral load or an increased titer of antibodies [117]. The avidity of IgG antibodies against the SARS-CoV-2 RBD domain was relatively low in most sera collected from patients within two months after the onset of symptoms, with a slight increase over time [118]. It is suggested that the activation and differentiation of memory B cells and the formation of antibody-secreting B cells (i.e., plasmablasts and plasma cells) may be absent and asynchronous in recovered subjects [34]. An efficient humoral immune response does not occur in every infected person, and no correlation exists between B cell levels and virus-specific IgG antibodies in peripheral blood.

Theoretically, vaccination should lead to the formation of immune protection against key SARS-CoV-2 antigens. Given that sterile immunity is not formed against SARS-CoV-2 infection and reinfection is possible, the problem of vaccination efficacy is related not so much to the COVID-19 vaccines themselves as to the nature of the immune response to this infection [34,35,119].

### 3.7. Specific IgG4 Antibodies During COVID-19 Vaccination

The mechanism of the adaptive immune response in the context of viral infection or following vaccination is conditioned by the subclasses of human IgG1 and IgG3 antibodies, which can stimulate immune cells through the activation of Fcγ receptors (FcγR) and the complement system activation pathway initiated by C1q [120,121]. The IgG1 and IgG3 antibody subclasses can form hexamers, facilitating interaction with the six-chain C1q molecule [120,122]. Ultimately, the IgG1 and IgG3 effector activity is due to antibody-dependent cell-mediated cytotoxicity (ADCC) and antibody-dependent cellular phagocytosis (ADCP). In this case, T- and B-memory cells are formed during the inflammatory response to infection, which triggers the synthesis of specific antibodies upon repeated contact with the infectious agent [120,121].

The formation of an immune response to a new coronavirus infection remains largely ununderstood. Clinical studies examining vaccine efficiency primarily focus on assessing the neutralizing activity of specific antibodies. Vaccine efficiency decreases over time, possibly due to a switch in the humoral immune response to specific IgG4 antibody synthesis. A long-term study of the change in specific antibodies during vaccination found a short-term predominance of IgG1 and IgG3 antibodies after immunization, which lasted approximately six months, followed by a decrease in their levels. From the 7th month, vaccinated patients begin to synthesize specific IgG4 antibodies [10,13,122,123]. Moreover, specific IgG4 synthesis is observed in response to the administration of the mRNA vaccines.

Unlike pro-inflammatory IgG1 and IgG3 antibodies, IgG4 antibodies cause anti-inflammatory effects, mainly due to their affinity for the classical inhibitory IgG receptor FcγRIIB [121,124,125]. IgG4 antibodies cannot activate C1q. Moreover, they inhibit the formation of hexamers of IgG subclasses that activate the complement C1q [120]. In addition, IgG4 is capable of exchanging Fab arms, allowing heavy chains with different specificities to dimerize and form bispecific antibodies, thereby reducing their ability to form immune complexes [126]. Thus, IgG4 antibodies can block IgG1 antibodies or IgE inflammatory effects, expelling the binding of antibodies with comparable specificity [127].

The literature includes only rare reports of the switch in the immune response to IgG4 synthesis after an infection or vaccination, except in the following examples. A study assessing the post-infectious immune response to Plasmodium falciparum (the parasite causing malaria) among children in Mozambique found that IgG3 reactivity predominated in the first two years after infection, whereas IgG4 reactivity was low. In two years, children exhibited high concentrations of neutralizing IgG1 and IgG3, which were associated with a reduced probability of acquiring malaria by the second year of life. The doubling of IgG1 levels was associated with a 50% reduction in the risk of malaria infection. At the same time, the likelihood of malaria infection increased approximately threefold with the doubling of IgG4 levels [128]. Additionally, IgG4 was shown to prevent the opsonization of infected IgG1 and IgG3 erythrocytes in vitro [129]. Another study revealed an association between high IgG4 levels and an increased risk of malaria infection and exacerbation [130]. During anti-HIV vaccination (seven rounds of immunization with the VAX003 vaccine), the initiation of IgG4 synthesis was observed. IgG4 removal from the serum of vaccinated individuals resulted in a significant increase in Fc-mediated effector activity of the serum, attributed to the presence of IgG3 antibodies [131]. Assessment of acellular pertussis (aP) vaccines’ efficacy revealed the induction of IgG4 antibody synthesis, which is not observed with whole-cell (wP) pertussis vaccines. The authors considered that the protection efficacy of the pertussis vaccine, which involves the synthesis of IgG4 antibodies, remains lower than that with the synthesis of IgG1 antibodies [132,133].

A study of the mechanisms of switching the immune response to the IgG4 synthesis during allergic diseases with specific immunotherapy showed that in the repeated administration of the allergen, a change in the immune response to the antigen occurred with an increase in the concentration of the immunosuppressive cytokine IL-10, and in the level of Treg cells (CD25+Foxp3+CD4+), mediating mechanisms of tolerance to a specific antigen.

Thus, the mRNA vaccines initiate the long-term synthesis of IgG4 during COVID-19 vaccination. At the same time, in patients who had COVID-19 before immunization, no increase in IgG4 levels is observed, even after receiving the mRNA vaccine [122]. Clinical observations demonstrated that deceased patients with severe COVID-19 showed higher IgG4 levels than those who recovered, and the mortality rate increased notably after 30 days when the serum IgG4 concentration was above 700 mg/dL. The IgG4 to IgG1 ratio was above 0.05 [134]. Additionally, IgG4 levels correlate with IL-6 levels, a known risk factor for mortality associated with COVID-19 [135,136,137].

The features of IgG4 functional activity have been described over the past 20 years. Currently, IgG4 subclass antibodies are defined by the term “blocking antibodies,” associated with their reduced ability to mediate immune effector reactions [138,139]. That means when IgG4 antibodies interact with molecules, a “minimal” immune response develops [126]. At the same time, the IgG4 response can be either pathogenic or protective, depending on the case. In particular, IgG4 can suppress inflammation by competing with inflammatory IgE for antigen binding, as seen in allergies and infections caused by helminths and filarial parasites. Also, IgG4 antibodies can lead to severe manifestations in some autoimmune and cancer conditions [140,141].

Compared to BNT162b2, the mRNA-1273 vaccine can induce a prolonged IgG4 response. The amount and duration of spike protein produced seem to be affected by the higher concentrations of mRNA in the mRNA-1273 vaccine (100 μg) compared to the BNT162b2 vaccine (30 μg). Interestingly, among the mRNA vaccines, the mRNA-1273 vaccine induced elevated concentrations of anti-S1 serum IgG4 antibodies in individuals with no previous COVID-19 infection, with unknown consequences concerning protection against pathogens [123]. Among the IgG antibodies produced against the spike protein or its fragments, the level of IgG4 antibodies increased most noticeably (steadily from 0.04% right after the second vaccination to 19.27% after the third dose) [12].

Thus, further study of the immune response mechanism during the development stage of SARS-CoV-2 infection remains necessary. The assessment of changes in the production of specific IgG4 antibodies following booster vaccination remains of particular interest.

### 3.8. Vaccine Efficacy Against Respiratory Droplet- and Airborne Dust-Transmitted Infections

The development of vaccines against measles, mumps, and chickenpox (VZV) and their implementation in healthcare practice have radically reduced the incidence of these infectious diseases worldwide. However, not all vaccines against airborne infections demonstrate such a high level of long-term protection. In particular, anti-influenza vaccines offer short-term protection (less than a year) against new virus strains, and current generations of vaccines against respiratory syncytial virus (RSV) provide only weak protection [141,142,143,144,145,146,147,148,149,150].

COVID-19 vaccines have reduced the mortality rate. They have also prevented severe disease in a large number of patients suffering from a new coronavirus infection, thereby securing extensive control over the progression of the pandemic [141]. As new SARS-CoV-2 strains emerge, the protection efficacy of the original generation of vaccines against COVID-19 decreases. Long-term experience of vaccine use against influenza indicates complex issues with forming stable long-term protection immunity following immunization, both against seasonal and non-seasonal strains of the infection [151,152,153,154]. Influenza vaccination reduces the risk of the disease progressing to a more severe form and decreases hospital admissions and potential fatality, as with COVID-19 vaccines. The effectiveness of influenza vaccines against symptomatic disease (of any severity) has ranged from 10% to 60% in the last 20 years [155,156]. At the same time, the duration of immunity after vaccination is limited to several months, implying annual vaccination with new strain-type antigens. It should be noted that the actual levels of efficacy of influenza vaccines would be difficult to justify for approval of vaccines against other infections [154].

Comparing the characteristics of viral infections for which post-infection or post-vaccination immunity is characterized by long-term solid protection (measles, rubella, mumps, smallpox, chickenpox) with those for which the infectious process or immunization causes the formation of incomplete, relatively short-term protection (RSV, influenza, and COVID-19), the following differences can be highlighted. Viruses of the first group enter the mucous membranes of the respiratory tract, replicate in the mucosal epithelial cells, and then pass into the blood circulation, causing viremia when they contact various cells, including immunocompetent ones. These infections are also characterized by a prolonged incubation period (10 to 21 days), during which the virus particles replicate in the mucous membranes. Eventually, a strong, long-term, or lifelong immunity is established during the infection. At the same time, for infections with weak or incomplete post-immunization protection (RSV, influenza, and COVID-19), the incubation period is shorter (ranging from 2 to 5 days), and rapid viral replication occurs in the mucous membranes. In the case of COVID-19, the so-called “RNAemia” of SARS-CoV-2 (circulation of viral RNA in the blood) was revealed, similar to RNAemia in influenza [157,158,159,160]. Several authors associate the absence of stable protection for these infections with the high variability of the viruses [161]. At the same time, RSV is not characterized by high mutagenic activity, but long-term protective immunity does not emerge in patients who have recovered from the disease, which predetermines the possibility of multiple reinfections throughout the lifespan [162,163,164,165,166].

The peculiarities of RSV infection and the establishment of an immune response to RSV are related to the fact that the antiviral immune response develops along the Th2 pathway in infants during the first months of life. This is due to the typical neonatal immune profile characterized by increased secretion of Th2-mediated cytokines (IL-4, IL-5, and IL-10). This shift represents an evolutionary protection mechanism of a child before birth against the damaging effects of maternal bioactive cytokines of the Th1 immune response pathway, including interferons (IFNs). In elderly age, a shift towards the Th2 response appears again. RSV infection itself causes a Th1/Th2 imbalance to a much greater extent than other respiratory viruses, including influenza. This feature is the reason that the severity of acute diseases caused by RSV is often much more significant than that of acute respiratory viral infections (ARVI), and also that children who have had RSV infection in infancy are significantly more often diagnosed with bronchial asthma in older age [167].

## 4. Discussion

The short-term efficacy of COVID-19 vaccines and the emergence of new SARS-CoV-2 strains have stimulated the development and research of new vaccines in four directions. First, drugs are being adapted to new relevant strains of the novel coronavirus infection. Second, vaccines are being developed based on novel technology platforms and improvements to existing technologies. Third, new routes and administrative schemes are being developed. Fourth, due to the stabilization of the epidemic process and the need to integrate SARS-CoV-2 immunization into mass routine vaccination programs, the development of combination drugs that protect against COVID-19 and several other respiratory infections (e.g., seasonal influenza, seasonal beta-coronaviruses, RSV) is of particular interest.

Given the high mutational variability of the SARS-CoV-2 virus, research is ongoing to develop COVID-19 vaccines targeting current and anticipated virus strains. In particular, Moderna developed several bivalent booster immunization vaccines (mRNA-1273.351 (Beta), mRNA-1273.617 (Delta), mRNA-1273.529 (Omicron), mRNA-1273.211 (Wuhan strain + Beta), mRNA-1273.213 (Beta + Delta) and mRNA-1273.214 (Wuhan strain + Omicron) (NCT04927065). Pfizer and BioNTech also developed drug products based on the Beta, Delta, and Omicron variants [168]. Clinical trials of a booster vaccine adapted to new antigenic variants of coronavirus (Moderna-1273.214 (Wuhan strain + Omicron)) demonstrated higher immunogenic activity against the BA.1, BA.4, and BA.5 Omicron subvariants [15,169,170]. Among initially seronegative subjects, one month after booster administration, it was found that mRNA-1273.214 induced a 1.6-fold higher geometric mean titer (GMT) of neutralizing antibodies against Omicron than mRNA-1273 (based on the master strain)—2372 [95% CI: 2071, 2718] vs. 1473 [95% CI: 1271, 1708]. At the same time, GMT against BA.4 and BA.5 was three times lower than against BA.1.

The monovalent vaccine (based on Omicron) produced by Pfizer-BioNTech at different dosages (30 and 60 μg) induced 2.23- and 3.15-fold higher levels of NAb GMT against Omicron, respectively, compared to the original version of the drug product. The bivalent mRNA-1273.214 vaccine (based on two Omicron subvariants) at 30 and 60 μg produced 1.56 and 1.97-fold NAb GMT against Omicron compared to the original drug product, respectively [171]. At the same time, vaccines based on the BA.1 Omicron subvariant demonstrate a lower neutralizing activity against new SARS-CoV-2 Omicron strains. For example, the mRNA vaccine Moderna-1273.214 (Wuhan variant + Omicron) induced a neutralizing activity that was threefold lower against BA.4/5 than against BA.1. Similarly, a Pfizer vaccine study also found that in live virus neutralization tests serum from the subjects vaccinated with the first-generation Omicron strain-based drug product neutralized BA.4/5 three times less efficiently than BA.1. The data obtained confirm the assumption that the BA.4/5 sub-strain has acquired the property of “escaping” the influence of specific neutralizing antibodies induced by vaccines based on the BA.1 sublineage [172].

The other direction of new vaccine development remains primarily based on data demonstrating that the sera of people infected with SARS-CoV in 2003 exhibit neutralizing activity against SARS-CoV-2, which justifies the fundamental possibility of developing a universal pan-coronavirus vaccine against beta-coronaviruses [173,174,175,176]. In particular, Cohen A.A. et al. developed multivalent nanoparticles that mimic the RBD of eight different coronaviruses, named “Mosaic-8” [177]. In an animal study, immunization with this drug produced similar levels of neutralizing antibodies against different SARS-CoV-2 variants, including Omicron, and protected against the SARS-CoV-2 infection [177]. In addition, chimeric spike proteins can also induce pan-coronavirus NAbs. In particular, Martinez D.R. et al. created a construction consisting of chimeric spike proteins of the following types of sarbecoviruses: SARS-CoV, SARS-CoV-2, SARS-CoV-2 B.1.351, bat CoV (Bt-CoV) RsSHC014 and heterologous Bt-CoV WIV-1. An mRNA vaccine based on these antigens caused a more efficient neutralization of SARS-CoV-2 subvariants B.1.1.7 (from the UK) and B.1.351 (from South Africa) and multiple branches of sarbecoviruses than monovalent SARS-CoV-2 in mice [178]. The authors believe multiplex-chimeric spikes can prevent SARS-like zoonotic coronavirus infections with pandemic potential.

Research is being conducted to develop vaccines targeting conserved regions of the SARS-CoV-2 virus, which are hidden within the three-dimensional protein structure or encapsulated in the virus particle, making them difficult to bind and neutralize by antibodies. Long-term T-cell immunity in convalescent patients’ plasma taken approximately three years after SARS-CoV infection showed cross-reactivity with the SARS-CoV-2 N protein [179]. In particular, the nucleocapsid is added to the S protein in the vaccines developed by Gritstone and ImmunityBio [180]. Similarly, in the study by Ong E. et al., the spike protein (S), nucleocapsid protein (N), and membrane protein (M) were tested to develop vaccines against SARS and MERS. With the use of the Vaxign-ML (reverse vaccinology) machine learning approach, six proteins, including the S protein and five non-structural proteins (nsp3, 3CL-pro and nsp8-10) were shown to be adhesins critical for virus attachment and invasion. Based on the results of Vaxign-ML analysis, the authors suggest proteins S, nsp3, and nsp8 have a high protective potential [181]. Thus, non-structural protein antigens can also be used to create multivalent, multi-epitope peptide or mosaic COVID-19 vaccines [182,183].

## 5. Conclusions

The experience of mass vaccination during the COVID-19 pandemic suggests that new vaccines were developed successfully within a remarkably short period, marking a first-time achievement. This development became possible due to advances in biotechnology, genetics, and virology, as well as the emergence of new biotechnological platforms in recent decades. COVID-19 vaccines prevent the disease from increasing in severity; they reduce hospital admissions and fatality rates.

One of the first issues identified in clinical studies and during mass immunization campaigns was the short-term efficacy of COVID-19 vaccines (up to about six months) and the absence of sterile post-immunization protection. To assess and extend the duration of protective efficacy, epidemiological and clinical studies have begun to determine precise schedules for vaccine administration, as well as the number of primary series and booster doses. These studies showed a higher efficiency (in terms of immunogenicity) when using heterologous vaccines for prime and booster immunizations. At the same time, the high variability of the SARS-CoV-2 virus did not allow the issue of vaccine efficacy to be fully resolved by optimizing the booster regimens solely.

The following justified step was the development of vaccines based on actual strains of SARS-CoV-2, which we are currently seeing. Creating a consistent approach to promptly replacing “versions” of drugs that have been adapted to target current strains of the virus (e.g., influenza vaccines) is complicated by the fact that the levels of neutralizing antibodies needed to ensure protective efficacy (a correlate of protection) against different SARS-CoV-2 strains have not yet been determined. Additionally, studies have demonstrated that different SARS-CoV-2 strains can evade the action of neutralizing antibodies to varying degrees.

COVID-19 has become a year-round infection with a pronounced seasonal component. At the same time, the Omicron variant of SARS-CoV-2, which has been mainstream since the end of 2021, causes a milder course of the disease on average than previous variants. However, it is impossible to predict whether subsequent strains and substrains of SARS-CoV-2 will cause the development of more severe clinical symptoms, lead to higher mortality, and to what extent new strains will be able to evade the action of previously formed neutralizing antibodies. Therefore, when developing COVID-19 vaccines, it is necessary to determine and validate the immune correlates of protection using NAb titers or concentrations, as well as additional biomarkers, including indicators of cellular immunity. The roles and importance of viremia, avidity, and the types of specific antibodies in immune defense mechanisms in COVID-19 should also be evaluated.

Thus, on the one hand, COVID-19 is a new infection, and the mechanisms of its development have not yet been sufficiently understood in detail. Consequently, the advancement of highly efficacious vaccines demands further research into a multitude of factors, including potential virus mutations, the characteristics of inflammatory reactions, the systemic and local innate and adaptive immune responses to diverse strains of the coronavirus, and numerous other factors. On the other hand, the features of the clinical course of the disease and immune response to COVID-19 vaccination have some similarities with some respiratory viral infections, such as influenza, adenovirus, and RSV. It is therefore crucial to consider previous experience and established strategies for mass immunization, including the scientific and regulatory principles governing the periodic antigenic adaptation of vaccine products, when developing new approaches for preventing SARS-CoV-2 infection.

## Figures and Tables

**Table 1:** Efficacy of COVID-19 vaccines (examples).

Vaccines	Vaccine Type	Efficacy		References
		Vaccine Efficacy in Preventing COVID-19 Illness (Of Any Severity) (%)	Vaccine Efficacy in Preventing Severe COVID-19 Illness (%)	
Spikevac (mRNA-1273)	mRNA	93.2	98.2	[4]
Comirnaty (BNT162b2)	mRNA	91.3	91.3	[5]
Ad26.COV2.S	Virus vector	52.4	74.6	[6]
Vaxzevria (ChAdOx1 nCoV-19, AZD1222)	Virus vector	74.0	100	[25]
Covaxin (BBV152)	Inactivated	77.8	93.4	[26]
Covilo (BBIBP-CorV)	Inactivated	78.1	100	[27]
CoronaVac (PiCoVacc)	Inactivated	50.7–83.5	100	[28,29,30]
Nuvaxovid (NVXCoV2373)	Subunit	89.7–90.4	100	[31,32]
Convidecia (AD5-nCoV)	Virus vector	57.5	91.7	[33]

**Table 2:** Immunogenicity and efficacy of COVID-19 vaccines (examples).

Vaccines	Vaccine Type	Neutralizing Antibodies (Geometric Mean Concentration, BAU/mL)	Vaccine Efficacy (%)	Blood Draw Post-Vaccination (day)	Number of Patients (n)	References
Spikevac (mRNA-1273)	mRNA	654.3	94.1	43	45	[2,82]
Comirnaty (BNT162b2)	mRNA	361	95	28	15	[7,83]
Ad26.COV2.S	Virus vector	First injection: 277–321 Second injection: 827	66	71 71	24 24	[84]
Vaxzevria (ChAdOx1 nCoV-19, AZD1222)	Virus vector	161–193	60.3–90	28	72	[3,85]
Covaxin (BBV152)	Inactivated	247	72.5	21	42	[86]
Covilo (BBIBP-CorV)	Inactivated	282.7	79.34	21	32	[87]
CoronaVac (PiCoVacc)	Inactivated	65.4		28	118	[88]
Nuvaxovid (NVXCoV2373)	Subunit	3906	89.3	35	29	[89]
SCB-2019 (Clover)	Subunit	1810–3320	N/A	36	31	[90]

## Abbreviations

The following abbreviations are used in this manuscript:

ADCC	Antibody-Dependent Cellular Cytotoxicity
ADCP	Antibody-Dependent Cellular Phagocytosis
AMSTAR	A Measurement Tool to Assess Systematic Reviews
ARVI	Acute Respiratory Viral Infection
CDC	Centers for Disease Control and Prevention
CI	Confidence Interval
COV	Coronavirus
COVID	Coronavirus Disease
EBA	Early Bactericidal Activity
ELISA	Enzyme-Linked Immunosorbent Assay
EMA	European Medicines Agency
FDA	Food and Drug Administration
GMT	Geometric Mean Titer
GRADE	Grading of Recommendations, Assessment, Development, and Evaluations
HAI	Hemagglutination Inhibition Assay
HIV	Human Immunodeficiency Virus
ICP	Infection Control Protocol
IL	Interleukin
MERS	Middle East Respiratory Syndrome
ML	Machine Learning
MRNA	Messenger Ribonucleic Acid
OR	Odds Ratio
PMCID	PubMed Central Identifier
PMID	PubMed Identifier
PRISMA	Preferred Reporting Items for Systematic Reviews and Meta-Analyses
RBD	Receptor Binding Domain
RNA	Ribonucleic Acid
RSV	Respiratory Syncytial Virus
SARS	Severe Acute Respiratory Syndrome
TICO	Therapeutics for Inpatients with COVID-19
VOC	Variant of Concern
VZV	Varicella Zoster Virus
